# Srag Regulates Autophagy via Integrating into a Preexisting Autophagy Pathway in Testis

**DOI:** 10.1093/molbev/msaa195

**Published:** 2020-07-28

**Authors:** Yibin Cheng, Fengling Lai, Xin Wang, Dantong Shang, Juan Zou, Majing Luo, Xizhong Xia, Hanhua Cheng, Rongjia Zhou

**Affiliations:** 1 Hubei Key Laboratory of Cell Homeostasis, College of Life Sciences, Wuhan University, Wuhan, China; 2 Renmin Hospital of Wuhan University, Wuhan University, Wuhan, China

**Keywords:** autophagy, new gene, reproduction, development, gene network

## Abstract

Spermatogenesis is an essential process for producing sperm cells. Reproductive strategy is successfully evolved for a species to adapt to a certain ecological system. However, roles of newly evolved genes in testis autophagy remain unclear. In this study, we found that a newly evolved gene *srag* (Sox9-regulated autophagy gene) plays an important role in promoting autophagy in testis in the lineage of the teleost *Monopterus albus*. The gene integrated into an interaction network through a two-way strategy of evolution, via Sox9-binding in its promoter and interaction with Becn1 in the coding region. Its promoter region evolved a *cis* element for binding of Sox9, a transcription factor for male sex determination. Both in vitro and in vivo analyses demonstrated that transcription factor Sox9 could bind to and activate the *srag* promoter. Its coding region acquired ability to interact with key autophagy initiation factor Becn1 via the conserved C-terminal, indicating that *srag* integrated into preexisting autophagy network. Moreover, we determined that Srag enhanced autophagy by interacting with Becn1. Notably, *srag* transgenic zebrafish revealed that Srag exerted the same function by enhancing autophagy through the Srag–Becn1 pathway. Thus, the new gene *srag* regulated autophagy in testis by integrated into preexisting autophagy network.

## Introduction

Spermatogenesis is a coordinated series of successive cellular processes that finally produces functional sperm cells. In which, main cellular events include mitosis of primordial germ cells and spermatogonia, meiosis from spermatocytes to spermatids, and differentiation of haploid spermatids to sperm cells in seminiferous tubules (Griswold 1998). Unlike other cellular processes, both mitosis and meiosis are necessary for spermatogenesis process. Over half of the murine genome was expressed during testis development (Shima et al. 2004). An average number of expressed genes in a single germ cell in human fetus was 7,510 based on single-cell sequencing analysis (Li et al. 2017). Mutations of some of these genes interrupted spermatogenesis and were associated with male infertility (Croft et al. 2018; Gonen et al. 2018; Krausz and Riera-Escamilla 2018). However, regulatory mechanisms of spermatogenesis are not well understood.

Autophagy is a catabolic process that is essential for cells to maintain homeostasis in various eukaryotes from yeasts to humans (Luo et al. 2016). Under physiological and pathological stress responses, autophagy can degrade intracellular undesired macromolecules/organelles for recycling and architectural remodeling by autophagosome–lysosome pathway via lysosomal hydrolysis (Yang and Klionsky 2010; Klionsky et al. 2016), which is essential for differentiation and development. Several studies have indicated that autophagy genes regulated spermatogenesis through maintaining homeostasis during spermatogenic cell differentiation, for example, *Atg5* and *Atg7* (Gao et al. 2018). These mutations eventually caused male infertility. As supporting cells, the Sertoli cells and Leydig cells provide proper microenvironment for germ cells during spermatogenesis. Accumulated evidence indicated that autophagy, as a mechanism for cell quality control in the supporting cells, safeguarded nearly all processes of spermatogenesis, and deficiency of autophagy in testis will disrupt spermatogenesis and cause male infertility. Given the complexity of spermatogenesis, roles and molecular mechanisms of autophagy regulations in the spermatogenesis process remain largely elusive.

In recent years, newly evolved genes in some organisms have been identified. These new genes only presented in a subset of species in a phylogeny during evolution at a locus that did not exist previously (Chen et al. 2013). The new genes were lineage- and/or species-specific and evolved important functions. Accumulated evidence suggested that new genes were implicated in development in diverse taxa ranging from *Drosophila* to mammals, particularly in brain functions in humans (Charrier et al. 2012; Dennis et al. 2012) and male reproductive development in *Drosophila* (Lee et al. 2019; Witt et al. 2019) and mammals (Chen et al. 2013). For example, testis specifically expressing *Poldi* was a recently emerged new gene in mice. Knockout of the gene led to reduced testis weight and sperm motility (Heinen et al. 2009). Sdic family genes were recently originated in *Drosophila*, which were essential for sperm competence (Yeh et al. 2012). New genes tend to be expressed in the testis, indicating that testis is a hotspot for birth of new genes due to rapid evolution of new sequences, structures, and expression under positive selection (Long et al. 2003). However, how new genes tend to be biased toward the testis and rapidly become essential for spermatogenesis remain unclear.

In addition to economic importance as a freshwater fish in aquaculture production, the teleost *Monopterus albus* is an increasingly recognized model species for development, genetics, and evolution (Cheng et al. 2003). It has attractive feature of natural sex reversal from female via intersex into male during its life (Liu 1944), with a peculiar theoretic significance in sex determination (Bullough 1947). This reproductive advantage ensures a successful establishment of new colonies in isolated small populations. Along with its air-breath ability, these features make the species a successful invader around the globe. In fact, it has the potential for disrupting currently threatened ecosystems (Collins et al. 2002). Furthermore, its genome is small (∼800 Mb), and among the teleosts, it has the smallest haploid number (*n *=* *12) of chromosomes (Zhou et al. 2001). Recently, we have sequenced the *Monopterus* genome (Zhao et al. 2018). It diverged from the related teleost fishes (tilapia, medaka, and platyfish) ∼70.3 Ma, indicating its recent origin within the teleosts. This invites us to identify new genes in the lineage of the *Monopterus* for its reproduction advantage, which would provide new insights into mechanisms of sexual differentiation and gametogenesis.

In this report, we observed that autophagy was upregulated in testis compared with ovary and ovotestis in the *Monopterus*. By genomic approach, we identified a newly evolved gene *srag* from the *Monopterus* genome, which was highly expressed in testis in comparison with both ovary and ovotestis in the species. Both in vitro and in vivo analyses demonstrated that Sox9, a transcription factor for male development, could bind to and activate the *srag* promoter, suggesting a newly evolved *cis* element for binding of Sox9. Transgenic zebrafish revealed that Srag exerts its function by enhancing autophagy. Furthermore, we determined that Srag enhanced autophagy by interacting with key autophagy initiation factor Becn1. Our research demonstrated that the new gene *srag* evolved a novel function by integrated into preexisting gene network in autophagy regulation in testis.

## Results

### Upregulation of Autophagy in Testis

**FIG. 1.**
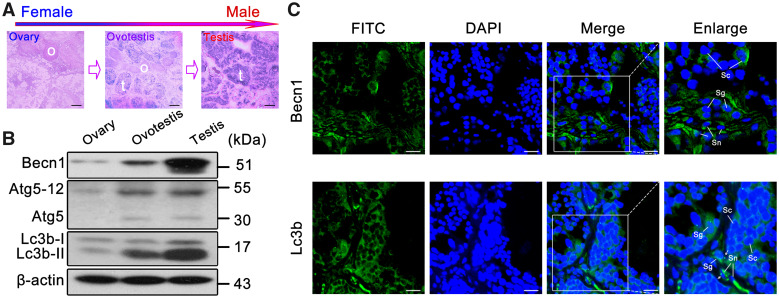
Upregulation of autophagy in testis. (*A*) H&E staining of the gonad tissues in *Monopterus albus*. The gonad transforms from ovary (o) to testis (t) via ovotestis during sex reversal. Scale bar: 100 μm. (*B*) Western blot analysis showed that Becn1, Atg5–Atg12, and Lc3b-II were upregulated during gonad transition. β-Actin was used as an internal control. (*C*) Immunofluorescence of the Lc3b and Becn1 proteins in testis using the anti-LC3B and anti-BECN1 antibodies followed by FITC-conjugated ImmunoPure goat anti-rabbit IgG (green). Lc3b was mainly expressed in the cytoplasm of Sertoli cells (Sn), spermatogonia (Sg), and spermatocytes (Sc) in testis. Lc3b-II puncta were detected in Sertoli cells and spermatogonia in testis (white arrows). Becn1 was expressed in Sertoli cells, spermatogonia, and spermatocytes in testis. The nuclei were revealed using DAPI fluorescence (blue). Images were captured using confocal microscopy. The enlarged images originated from the white squares. Scale bar: 10 μm.

To investigate whether autophagy is involved in gonad development, we examined protein levels of key autophagy regulators in testis, ovotestis, and ovary. Western blots showed that key autophagy regulators Lc3b-II (lipidated form of the autophagy protein Lc3b), Becn1, and Atg5–12 were upregulated in testis in comparison with ovotestis and ovary (fig. 1*A* and *B*). Immunofluorescent analysis indicated that both Lc3b and Becn1 were mainly expressed in the cytoplasm of Sertoli cells, spermatogonia, and spermatocytes in testis, and Lc3b-II puncta appeared in Sertoli cells and spermatogonia (fig. 1*C*). These results suggested that autophagy was upregulated in testis.

### Identification of New Genes with Testis-Biased Expression

**FIG. 2.**
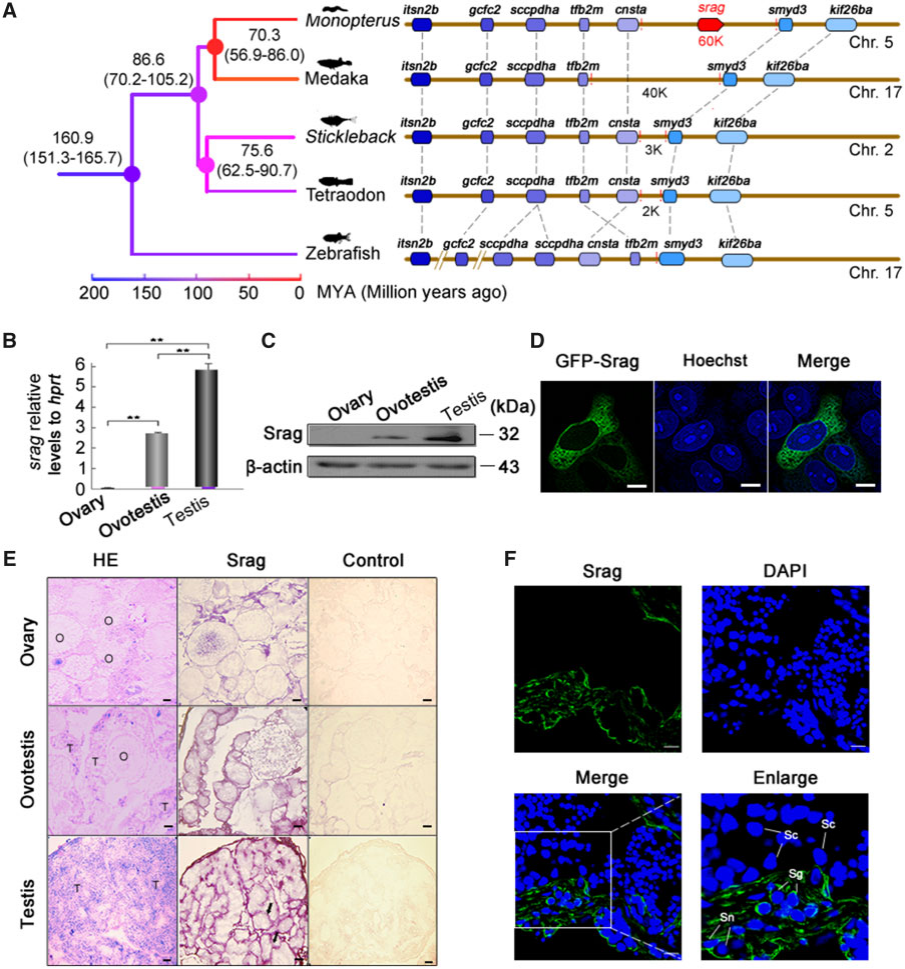
Identification and expression of *srag* in testis. (*A*) Synteny analysis. New gene *srag* in the genome of *Monopterus* has not any homologous genomic region in other teleost species. *srag* is a new gene on chromosome 5 of *Monopterus*; other genes on the chromosome are syntenic and conserved between *Monopterus* and other fishes (medaka, *stickleback*, tetraodon, and zebrafish). The number on the nodes indicates the time ranges of divergence from the present (Ma). (*B*) Quantitative real-time PCR of *srag* in gonads of *Monopterus*. Total RNA samples were isolated from ovary, ovotestis, and testis of adult individuals. *srag* mRNA expression was upregulated from ovary to testis. *hprt* was used as an internal control. (*C*) Western blot analysis showed Srag protein levels in different gonads using the anti-Srag antibody. β-Actin was used as an internal control. (*D*) Srag was expressed in the cytoplasm of HeLa cells after transfection. Images were captured using confocal microscopy. The nuclei were revealed using Hoechst fluorescence (blue). Scale bar: 5 μm. (*E*) Expression analysis of *srag* by mRNA in situ hybridization on gonad sections in adult individuals. Positive signals were observed in peripheral region of seminiferous epithelium (arrows). H&E staining indicates tissue structure of gonads. Labeled sense-strand DNA was used as a control. Scale bar: 50 μm. (*F*) Immunofluorescent localization of Srag protein in gonads using anti-Srag antibody followed by FITC-conjugated ImmunoPure goat anti-rabbit IgG (green). Srag was expressed in the cytoplasm of Sertoli cells (Sn) and spermatogonia (Sg) in testis. The nuclei were stained by DAPI (blue). Images were captured using confocal microscopy. The enlarged images originated from the regions with white squares. Scale bar: 10 μm.

To investigate key genes to regulate autophagy in testis, we developed a pipeline to screen new genes which emerged in the *Monopterus* lineage. All vertebrate and invertebrate protein sequences (64,393,748) from public databases were searched against the *Monopterus* protein sequences (20,456) by BlastP. Based on no significant BlastP hits in all organisms, excluded either too short genes or the genes without start and stop codons, 1,950 genes were determined as the candidate orphan genes. Of which, 1,533 were protein-coding genes, after confirmed by RNA-seq data sets (supplementary fig. S1, Supplementary Material online). We examined expression levels of the orphan genes in gonads using RNA-seq data. Comparisons of expression levels among three stages of gonads revealed an interesting pattern. In general, expression levels of orphan genes were significantly higher in testis than ovary and ovotestis (supplementary fig. S2*A*–*C*, Supplementary Material online). Of 1,533 orphan genes, reverse transcription-polymerase chain reaction (RT-PCR) of multi-tissues showed that 11 genes were gonad-biased, and five of them were highly expressed in testis. We then focused on one of new genes with testis-biased expression (supplementary fig. S2*D*, Supplementary Material online), which was tentatively named as *srag* (Sox9-regulated autophagy gene). Genomic alignments showed that no homologous sequence of *srag* was detected in genomes of other relative species (fig. 2*A*). Quantitative real-time PCR analysis indicated that *srag* was expressed only in ovotestis and testis, with high expression in testis (fig. 2*B*). Western blots showed that a major protein of 32 kDa was expressed mainly in testis and a slightly weak expression in ovotestis (fig. 2*C*). Obvious localization of Srag protein was in the cytoplasm of HeLa cells after transfection (fig. 2*D*). mRNA in situ hybridization showed that *srag* was expressed in peripheral region of seminiferous epithelium (fig. 2*E*). In addition, immunofluorescent analysis indicated that Srag protein was expressed in the cytoplasm of Sertoli cells and spermatogonia in testis (fig. 2*F*). These results suggested that the expression of new gene *srag* was testis-biased.

### Sox9 Regulates Spatiotemporal Expression of *srag* in Testis

**FIG. 3.**
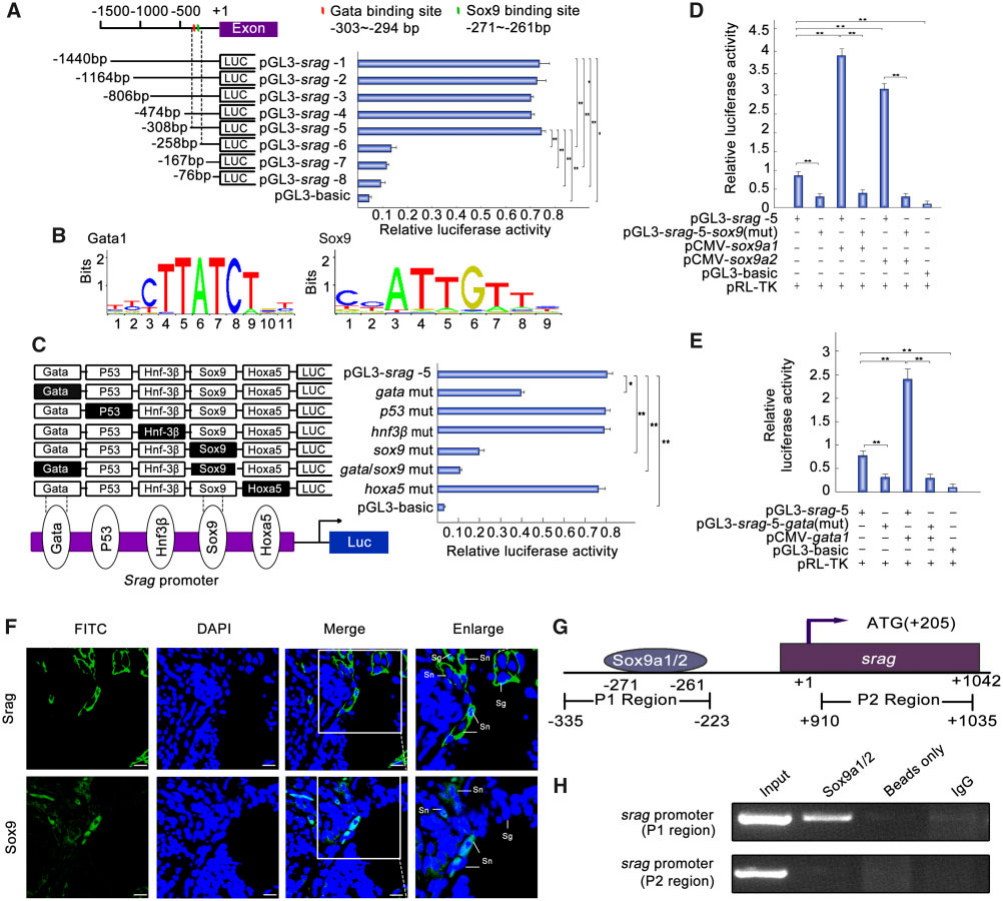
Sox9a1/2 and Gata1 upregulates *srag* promoter activity. (*A*) Luciferase assay indicated the activities of a series of truncated *srag* promoters in 293T cells. Endogenous SOX9 is expressed in 293T cells. Left panel showed each truncated mutant linked with the luciferase gene in the pGL3-basic vector. Binding sites of Gata and Sox9 were indicated in red and green bars, respectively. Right panel indicated the relative activities of these constructs, as determined by luciferase assays. (*B*) Sequence logo of Sox9- and Gata1-binding sites based on JASPAR database. (*C*) Point mutation analysis of the promoter using luciferase assays. The pGL3-*srag*-5 of 308 bp was used as a template for the point mutation analysis. Luciferase assays were used to determine the relative activities. The intact binding sites of the Gata, P53, Hnf3β, Sox9, and Hoxa5 are indicated by open boxes, respectively. The filled boxes show the corresponding mutations. The pGL3-basic vector was used as a negative control. (*D*) Sox9a1/2 overexpression upregulated the luciferase activity of *srag* promoter. *Sox9a1/2* transfection activated the *srag* promoter. In total, 0.32 μg pGL3-*srag*-*5* or its Sox9-binding site mutant (pGL3-*srag*-*5*-*sox9*(mut)) was cotransfected with 0.08 μg *sox9a1/2* expression plasmids (pCMV-*sox9a1* and pCMV-*sox9a2*), as indicated. Sox9a1 and Sox9a2 overexpression increased the activity of pGL3-*srag*-*5* but did not affect the activity of pGL3-*srag*-*5*-*sox9*(mut). (*E*) Gata1 overexpression upregulated the luciferase activity of *srag* promoter. Gata1 transfection activated the *srag* promoter. In total, 0.32 μg pGL3-*srag*-*5* or its Gata-binding site mutant (pGL3-*srag*-*5*-*gata* (mut)) was cotransfected with 0.08 μg Gata1 expression plasmids (pCMV-*gata1*). Gata1 overexpression increased the activity of pGL3-*srag*-*5*, but did not affect the activity of pGL3-*srag*-*5*-*gata* (mut). (*F*) Coexpression analysis between Srag and Sox9. Immunofluorescence of testis samples in serial sections using anti-Srag or anti-Sox9 antibody followed by FITC-conjugated ImmunoPure goat anti-rabbit IgG (green). Sox9 was expressed in the nuclei of Sertoli cells (Sn) in testis, and Srag was detected mainly in the cytoplasm of Sertoli cells (Sn) and spermatogonia (Sg). The nuclei were stained by DAPI (blue). Images were captured using confocal microscopy. The enlarged images originated from the regions with white squares. Scale bar: 10 μm. (*G*) Schematic diagram of primer relative positions in the ChIP assays. (*H*) ChIP assays. ChIP analysis showed that Sox9 could bind to the *srag* promoter in vivo. Sonicated chromatin from testis of the adult individuals was used for PCR amplification. A 112-bp fragment corresponding to the −335 to −223 region of the *srag* promoter was amplified using the immunoprecipitated DNA as a template which was immunoprecipitated with monoclonal anti-Sox9. In controls (no antibody [beads only] or preimmune IgG), no band from the anti-Sox9 antibody precipitates was observed. Exon of *srag* was used as a negative control. The means ± SD are from three independent experiments. One-way ANOVA was performed. *P < 0.05; **P < 0.01.

To identify the regulatory elements of *srag* expression in testis, a series of truncated potential promoters of *srag* were used to drive luciferase gene expression and luciferase activity was determined. The results showed that the sequence from −308 to −258 bp in the 5′-flanking region was essential to *srag* transcriptional activity (fig. *3A*). A Gata-binding site, a P53-binding site, an Hnf3β-binding site, an Sox9-binding site, and an Hoxa5-binding site were detected in the promoter region. To identify roles of these sites, site-directed mutants were constructed using wild-type pGL3-*srag*-*5* plasmid as the template. Compared with the wild-type pGL3-*srag*-*5* construct, the single Gata-binding site mutant showed a slight decrease in transcription activity; Sox9-binding site mutant showed an obvious decrease in promoter activity, with particularly low activity observed in the mutant of double Gata1- and Sox9-binding sites (fig. 3*B* and *C*). These results indicated that the binding sites for the transcription factors Gata1 and Sox9 are important for *srag* promoter activity. A luciferase reporter analysis was used to determine the binding of the transcription factors Gata1, Sox9a1, and Sox9a2 to the Gata-binding site and Sox9-binding site, respectively. *Sox9a1* and *Sox9a2* are two similar copies of *Sox9* genes in the *Monopterus* genome (Zhou et al. 2003). The analysis showed that Sox9a1 and Sox9a2 could significantly increase pGL3-*srag*-*5* luciferase activity. By contrast, the activity of the Sox9-binding site mutant was not upregulated by Sox9a1 or Sox9a2 (fig. 3*D*) Moreover, mutations in the Gata1-binding site decreased the *srag* promoter activity, and Gata1 could significantly increase pGL3-*srag*-*5* luciferase activity, whereas the activity of the Gata-binding site mutant was not upregulated by Gata1 (fig. 3*E*). As parallel controls, the same site-directed mutagenesis was used, with the wild-type pGL3-*srag*-*1* plasmid as the template, and the regulation trend was similar to the pGL3-*srag-5* mutagenesis (supplementary fig. S3*A* and *B*, Supplementary Material online). These results showed that the Sox9-binding site in the *srag* promotor is key for *srag* transcription.

Sox9 is essential to differentiation of Sertoli cells and sex determination in mammals (Chaboissier et al. 2004; Croft et al. 2018). Assuming Sox9 regulated expression of *srag* in Sertoli cells in *M. albus*, we investigated whether Sox9 is expressed in Sertoli cells and upregulated in testis as *srag* does. RT-PCR and western blot analysis indicated that Sox9a1/2 expression was upregulated in testis (supplementary fig. S4, Supplementary Material online). In addition, immunofluorescent analysis showed that Sox9a1/2 was also expressed in the nuclei of Sertoli cells in testis, in comparison with germ cell marker Vasa (supplementary fig. S4*C*, Supplementary Material online). Further immunofluorescent analysis showed that Sox9a1/2 and Srag were coexpressed in the Sertoli cells in testis; in addition, Srag was detected in the cytoplasm of spermatogonia (fig. 3*F*). Chromatin immunoprecipitation (ChIP) was performed to investigate whether the transcription factors Sox9a1/2 bind to the *srag* promoter of adult testis in *Monopterus* in vivo. A 112-bp DNA region from anti-Sox9 precipitates was amplified by PCR. Sequencing analysis confirmed that the amplified fragments were *srag* promoter-specific. Another DNA fragment in a distinct genomic region (exon) was amplified as a control to exclude the possibility of nonspecific binding to the *srag* promoter region, and no band from the anti-Sox9 antibody precipitates was observed (fig. 3*G* and *H*). These results indicated that Sox9a1/2 can specifically bind to the *srag* promoter region in vivo and regulate a spatiotemporal expression of *srag* in the Sertoli cells in testis.

### Srag Promotes Autophagosome Formation

**FIG. 4.**
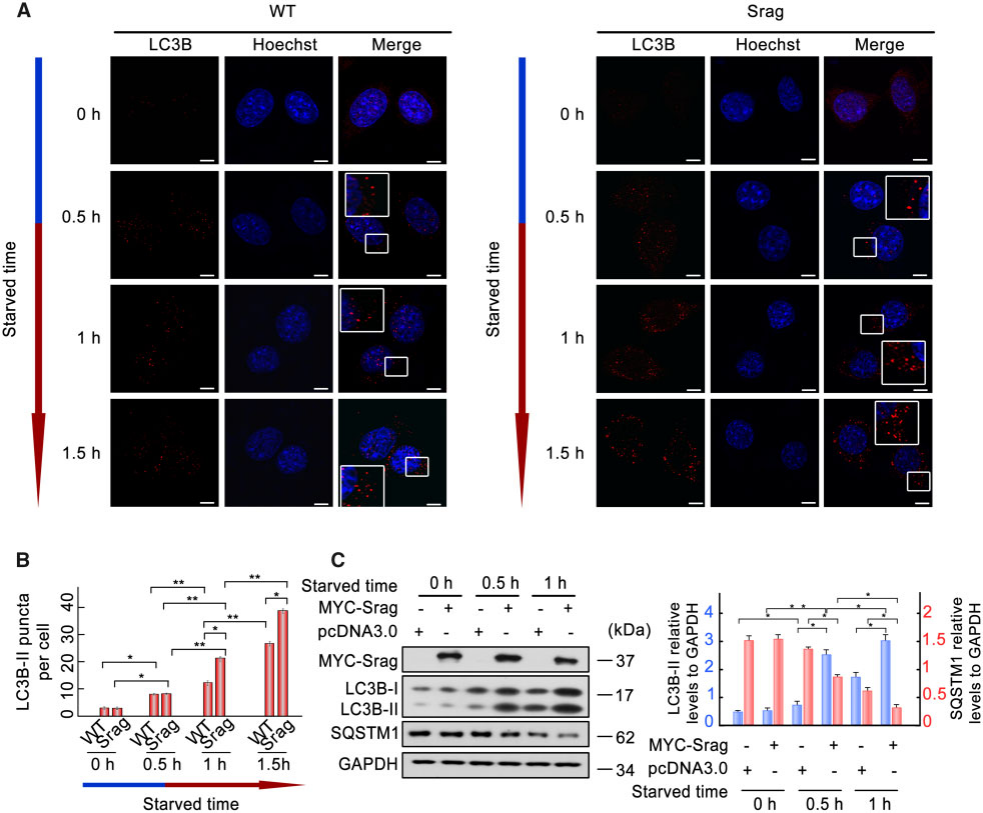
Srag overexpression promotes autophagy upon starvation induction. (*A*) Srag overexpression upregulated number of LC3B-II puncta. LC3B-II puncta were detected in wild-type or Srag overexpression HeLa cells cultured in the HBSS (Hanks balanced salt solution) medium for 0, 0.5, 1, and 1.5 h, respectively. Immunofluorescence was analyzed with anti-LC3B antibody and followed by confocal microscopy. LC3B-II puncta were detected in the cytoplasm by TRITC-conjugated ImmunoPure goat anti-rabbit IgG (red). The nuclei were revealed using Hoechst fluorescence. Enlarged boxes highlight LC3B-II signals. Scale bar: 5 μm. (*B*) Statistics of the LC3B-II puncta. The number of LC3B-II puncta was quantified from ∼20 cells for each group. The means ± SD are from three independent experiments. One-way ANOVA was performed. *P < 0.05; **P < 0.01. (*n* = 3 independent experiments). (*C*) *srag* overexpression upregulates LC3B-II level under starvation condition. HEK293T cells were transfected with equal amount of MYC-Srag or vector pcDNA3.0 (control) and cultured in the EBSS (Earle’s balanced salts solution) medium for 0, 0.5 and 1 h, respectively. Western blot analysis indicated that LC3B-II is upregulated by Srag overexpression in a time-dependent manner under starvation condition, whereas its downstream substrate SQSTM1 was downregulated. Cell lysates were analyzed by western blotting with the anti-LC3B and anti-MYC antibodies. GAPDH was used as an internal control. Western blots were quantified for LC3B-II/GAPDH and SQSTM1/GAPDH ratio. Data are presented as means ± SD. *P < 0.05; **P < 0.01 (*n* = 3 independent experiments).

As a similar expression pattern of Srag with key autophagy proteins (Lc3b and Becn1) in testis, *srag*-overexpressing HeLa cell lines were constructed to detect autophagosome formation under starvation condition. LC3B (microtubule associated protein 1 light chain 3 beta)-II puncta were detected under starvation culture condition in both *srag*-overexpressing and wild-type cells; however, the number of LC3B-II puncta was increased in *srag*-overexpressing cell lines, compared with wild-type (fig. 4*A* and *B*), indicating that Srag promoted autophagosome formation. To test its potential role in autophagy, *srag* was overexpressed in HEK293T cells, and the cells were treated in starvation medium to induce autophagy. Western blot analysis showed that LC3B-II level was upregulated (*P* < 0.05) upon starvation induction in *srag*-transfected cells in comparison with controls, whereas its downstream substrate SQSTM1 (sequestosome 1) was downregulated (*P* < 0.05) (fig. 4*C*).

### Srag from *Monopterus* Functions in Autophagy Regulation in Zebrafish

**FIG. 5.**
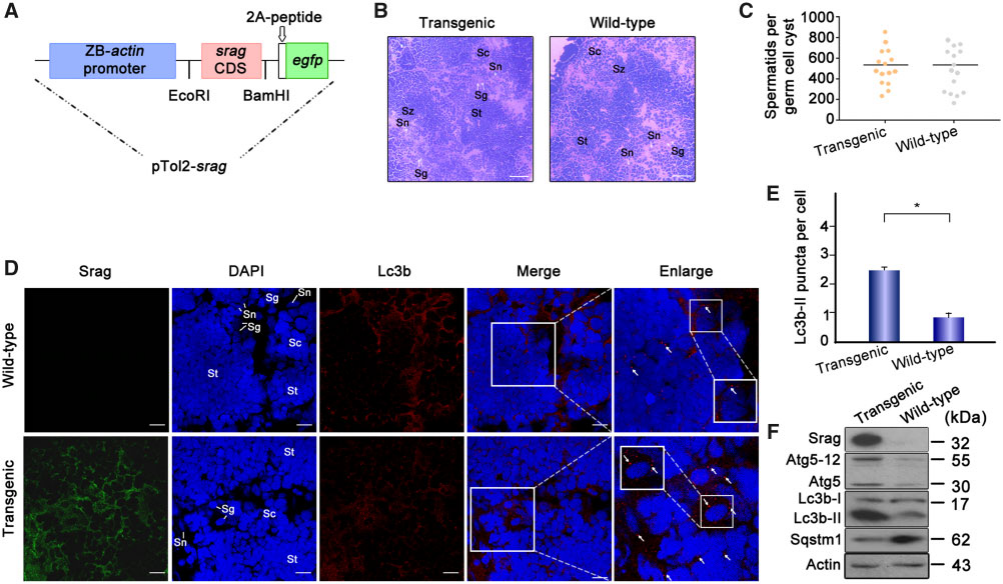
*srag* transgenic zebrafish analysis. (*A*) Schematic depiction of the pTol2-*srag*-*egfp* transgenic construct. The transgenic structure contains a region of the zebrafish *actin* promoter linked with the *srag* CDS (coding DNA sequence), a *gfp* tag, and 2A peptide cloned into the pTol2 vector. (*B*) Histological analysis of adult testis samples of *srag* transgenic and wild-type zebrafish by H&E staining. The white arrows indicate spermatogonia (sg) and Sertoli cells (sn). Scale bar: 50 μm. (*C*) Spermatid number in germ cell cyst in testis between *srag* transgenic and wild-type zebrafish (*P* < 0.9). (*D*) Immunofluorescence analysis of testis samples in both transgenic and wild-type zebrafish using anti-LC3B antibody. Images were captured using confocal microscopy. Lc3b-II puncta were obviously increased in the *srag* transgenic testis in comparison with wild type in spermatids (St), spermatocytes (Sc), and spermatogonia (Sg). The nuclei were stained by DAPI (blue). The enlarged images on the right panels originated from the white squares, the white arrows indicate Lc3-II puncta in spermatocytes (Sc). Enlarged box within the image highlights Lc3b-II puncta in Sertoli cells (Sn). Scale bar: 10 μm. (*E*) The number of Lc3b-II puncta was quantified from ∼20 cells for each group from (*D*). Data were presented as means ± SD. *T*-test was performed, *P < 0.05; **P < 0.01 (*n* = 3 independent experiments). (*F*) Western blot analysis of testis samples in both *srag* transgenic and wild-type zebrafish. Srag protein was detected in transgenic testis but not in wild type using the anti-Srag antibody. β-Actin was used as a control. Western blot analysis indicated that Lc3b-II and Atg5-Atg12 were upregulated, whereas Sqstm1 was downregulated in testis of transgenic zebrafish.

To further investigate the function of Srag in autophagy in vivo, *srag*-transgenic zebrafish lines were generated using the zebrafish *actin* core promoter-driven *srag* coding sequence (fig. 5*A*). Histological analysis showed no obvious morphologic change between adult testis samples of *srag*-transgenic and wild-type zebrafish (fig. 5*B*), and spermatid number per germ cell cyst was nearly same in transgenic and wild-type testes (fig. 5*C*), suggesting no morphologic change when *srag* was overexpression in transgenic zebrafish. Immunofluorescent analysis indicated that Lc3b was primarily expressed in the cytoplasm of spermatogenic cells and Sertoli cells in both transgenic and wild-type testes (fig. 5*D*). However, the number of Lc3b-II puncta was obviously increased in the *srag*-transgenic testis in comparison with wild-type (fig. 5*E*). Western blot analysis confirmed that Srag protein was expressed in the transgenic testis. Notably, Lc3b-II and Atg5–12 were upregulated in the *srag*-transgenic zebrafish testis in comparison with the wild-type, whereas Sqstm1 was downregulated (fig. 5*F*). These data suggested that not only *srag* overexpression can upregulate testis autophagy but also *srag* from *Monopterus* can upregulate autophagy in testis of zebrafish.

### Srag-associated Autophagy Flux

**FIG. 6.**
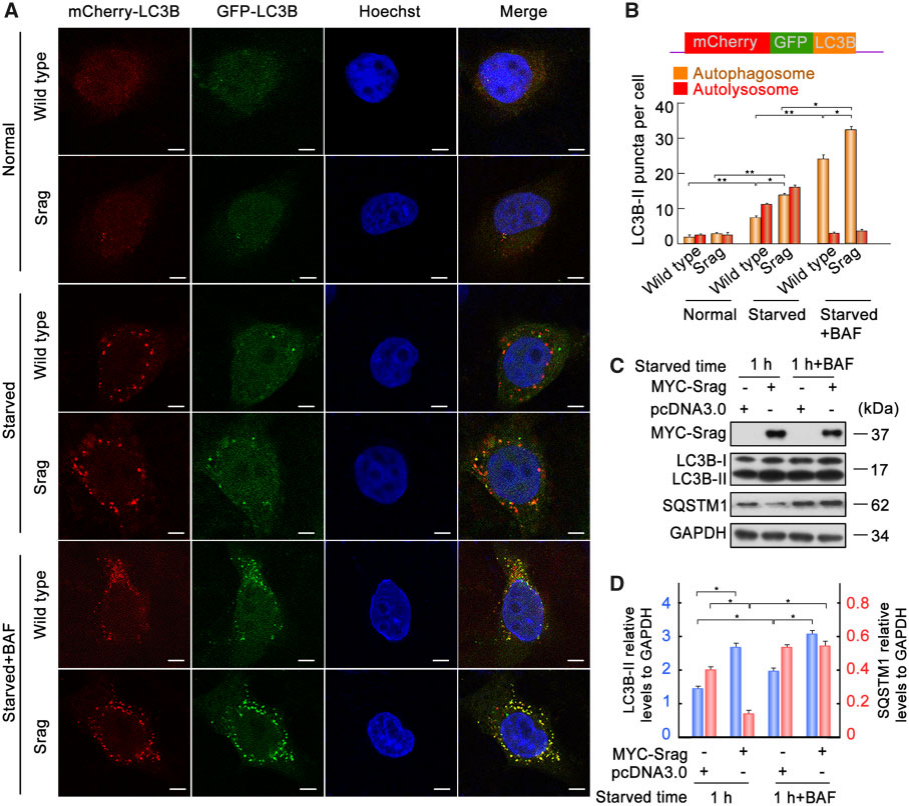
Srag-associated autophagy flux. (*A*) *srag* overexpressed and wild-type HeLa cells were transfected with the mCherry-GFP-LC3B tandem reporter. The cells were cultured in normal (control), EBSS medium (1 h), or EBSS with bafilomycin A1 (100 nM) addition (1 h), respectively. Single channel (red, green, or blue) and merged images were taken by confocal microscopy. (*B*) Statistic analysis of vesicles positive for both GFP and mCherry (autophagosomes) and for mCherry (autolysosomes) (>15 cells per experiment). Colocalized dots were counted. Data are presented as means ± SD. *P < 0.05, **P < 0.01 (*n* = 3 independent experiments). (*C*, *D*) *srag* overexpression promoted LC3B-II formation. Bafilomycin A1 treatment resulted in protein accumulation of LC3B-II. *srag* overexpression and control HEK293T cells were cultured and then starved in EBSS with or without bafilomycin A1 (100 nM) for 1 h. The cell lysates were analyzed by immunoblotting with the anti-LC3B, anti-SQSTM1, and anti-MYC antibodies. GAPDH was used as an internal control. (*D*) Western blots were quantified for LC3B-II/GAPDH and SQSTM1/GAPDH ratio. Data are presented as means ± SD. One-way ANOVA was performed, *P < 0.05; **P < 0.01 (*n* = 3 independent experiments).

To investigate Srag-associated autophagy process, we tested the Srag-involved autophagy flux through Srag-forced expression. Autophagy flux tests were performed using a tandem fluorescent indicator, mCherry-GFP-LC3B, in Srag-overexpressing HeLa cells and wild-type cells. Green fluorescence of the fusion protein is very sensitive in lysosomes and quickly quenched in autolysosomes, and yellow or green puncta indicate autophagosomes, whereas just red fluorescence could be detected in the autolysosomes (Sheng et al. 2018). Fluorescence analysis using the tandem fluorescent indicator system in these cell lines showed that *srag* overexpression promoted the autophagosome formation. Further bafilomycin A1 (an inhibitor for fusion of autophagosomes and lysosomes) treatment showed a significant accumulation of autophagosomes in *srag* overexpression (fig. 6*A* and *B*). In addition, western blot analysis showed that *srag* overexpression increased the LC3B-II level and decreased its downstream substrate SQSTM1 level upon starvation induction in HEK293T cells, and LC3B-II was accumulated in bafilomycin A1 treatment (fig. 6*C* and *D*). Together, these data demonstrated that Srag promotes formation of autophagosomes but does not affect fusion process of autophagosomes with lysosomes.

### Srag Interaction with Becn1 in Autophagy Regulation

**FIG. 7.**
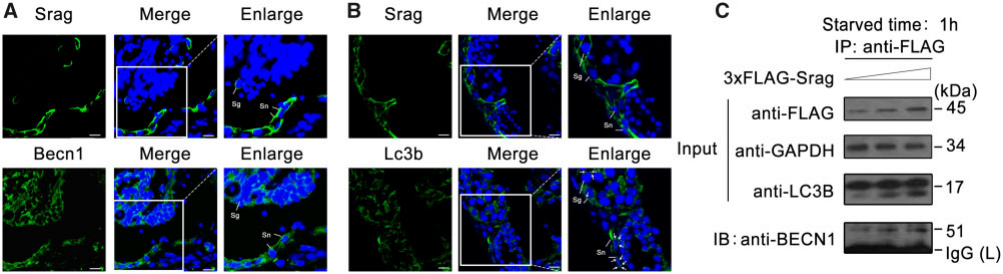
Srag coexpression with Becn1 and Lc3b and interaction between Srag and Becn1. (*A*, *B*) Coexpression analysis of Srag with Lc3b and Becn1. Immunofluorescence analysis of testis samples in serial sections using anti-Lc3b, anti-Srag, or anti-Becn1 antibody, respectively, then followed by FITC-conjugated ImmunoPure goat anti-rabbit IgG (green). The coexpression signals were detected in the cytoplasm of Sertoli cells (Sn) and spermatogonia (Sg) in testis. Lc3b-II puncta were detected in Sertoli cells and spermatogonia in testis (white arrows). The nuclei were stained by DAPI (blue). Images were captured using confocal microscopy. The enlarged images originated from the regions with white squares. Scale bar: 10 μm. (*C*) LC3B-II upregulation by *srag* overexpression and Co-IP between Srag and BECN1. HEK293T cells were transfected with an increasing amount of 3xFLAG-Srag (0, 0.5, and 1 μg). pcDNA3.0 was added for an equal amount DNA in each well. Western blot analysis indicated that LC3B-II is upregulated by Srag overexpression in a dose-dependent manner under starvation condition. Co-IP analysis indicated interaction between Srag and BECN1. Cell lysates were analyzed by western blotting with the anti-LC3B and anti-FLAG antibodies. GAPDH was used as an internal control.

**FIG. 8.**
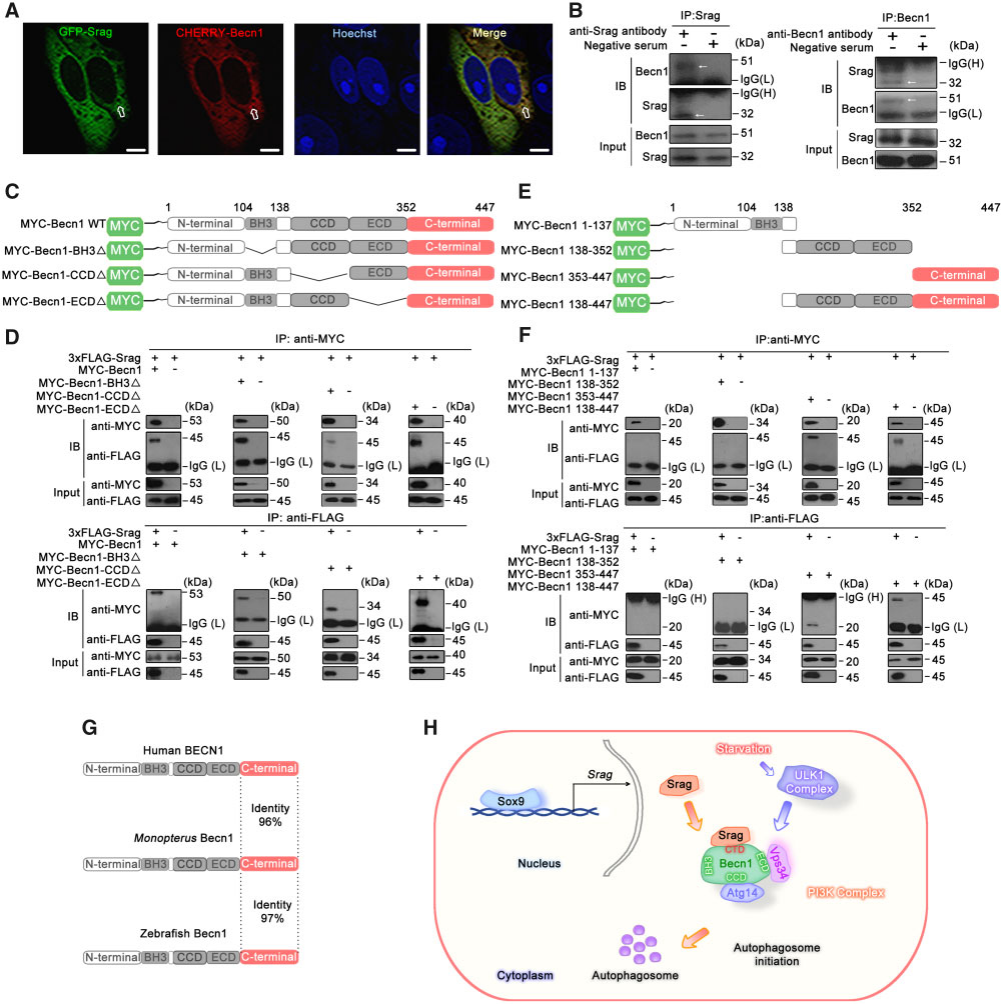
Srag interacts with Becn1. (*A*) Colocalization analysis between Srag and Becn1. HeLa cells were transiently cotransfected with GFP-Srag and mCherry-Becn1, followed by confocal microscopy. Colocalizing structures were indicated in yellow (merge). Arrows indicated the signals overlapped between Srag and Becn1. The nuclei were revealed using Hoechst fluorescence. Scale bar: 5 μm. (*B*) Co-IP analysis between endogenous Srag and Becn1 in testis of the adult *Monopterus*. The lysates were immunoprecipitated with anti-Srag, anti-Becn1 antibody, or negative serum, respectively, followed by immunoblotting with the anti-Becn1 or anti-Srag antibody, respectively. Arrows indicated the Co-IP band. (*C*) Schematic diagram of the *Monopterus* Becn1 wild-type and various deletions. The conserved domains (BH3, CCD, ECD, and C-terminal) are indicated in boxes. (*D*) Co-IP between Srag and deletion mutants of Becn1. 293T cells were transiently cotransfected with 3xFLAG-Srag and MYC-Becn1, MYC-Becn1-BH3Δ, MYC-Becn1-CCDΔ, or MYC-Becn1-ECDΔ. After transfection for 48 h, the whole-cell lysates were extracted for Co-IP with anti-FLAG or anti-MYC antibody, and anti-MYC or anti-FLAG antibody was used for western blot, respectively. The cell lysates were examined by western blotting using the anti-MYC or anti-FLAG antibody (input). (*E*) Schematic diagram of the *Monopterus* Becn1 truncated mutants. The conserved domains (BH3, CCD, ECD, and C-terminal) are indicated in boxes. (*F*) Co-IP analysis showed that Srag interacted with the C terminus of Becn1. 3xFLAG-Srag was cotransfected with MYC-Becn1 1–137, MYC-Becn1 138–352, MYC-Becn1 353–447, and MYC-Becn1 138–447 into 293T cells. The cell lysates were examined by western blotting using the anti-MYC or anti-FLAG antibody (input). The lysates were immunoprecipitated with the anti-FLAG or anti-MYC antibody, followed by immunoblotting with anti-MYC and anti-FLAG antibody, respectively. 3xFLAG-Srag can interact with MYC-Becn1 353–447 and MYC-Becn1 138–447. (*G*) Sequence alignments of the conserved C-terminal (pink) of BECN1 in human, *Monopterus*, and zebrafish. (*H*) Sox9–Srag–Becn1 pathway in autophagy regulation. *srag* promoter activity is activated by its transcription factor Sox9, which is bound to the *srag* promoter region in vivo. Srag interacts with the C-terminal of Becn1 to promote autophagy via PI3K complex upon starvation induction.

To further explore regulation mechanisms of Srag in autophagy, coexpression of Srag with autophagy regulator Becn1 in testis was first tested. Immunofluorescent analysis showed that Srag was colocalized with Becn1 in the Sertoli cells and spermatogonia in testis (fig. 7*A*). Immunofluorescence also showed that Srag was coexpressed with autophagy regulator LC3B in both Sertoli cells and spermatogonia; and Lc3b-II puncta formed in the cell types, Sertoli cells in particular (fig. 7*B*). Further co-immunoprecipitation (Co-IP) analysis indicated that Srag was associated with Becn1 upon starvation induction (fig. 7*C*). Fluorescent colocalization analysis confirmed that Srag was obviously colocalized with Becn1 in the cytoplasm in HeLa cells (fig. 8*A*). Co-IP from testis samples demonstrated that Becn1 could clearly interact with Srag in adult testis of *Monopterus* (fig. 8*B*). Further deletion and truncation mutation analysis showed that Srag can bind to the C-terminus of Becn1 but not to the BH3, CCD, or ECD domains (fig. 8*C*–*F*). In addition, the C-terminal region of Becn1 was evolutionarily conserved in vertebrates, including human, *Monopterus*, and zebrafish (fig. 8*G*). Taken together, these results suggested that Srag promotes autophagy through interaction with autophagy regulator Becn1, and the Srag has integrated into a preexisting network via Becn1 in autophagy regulation (fig. 8*H*).

## Discussion

Spermatogenesis is an essential process for successive generation to generation of a species. Mutations of key genes or disruption of involved pathways in the process will threaten species survival. Consequently, reproductive strategy should be successfully evolved for an organism to adapt to a certain ecological system. In this study, we have found that new gene *srag* plays an important role in enhancing autophagy. As an orphan gene, *srag* does not have recognizable homologs in other species, and it is testis-biased and a single exon gene, but it is probably not a retrogene or duplicated gene as no parental gene sequence has detected. Further study at population-level or in other closely related species could probably reveal its origination. Importantly, this new gene integrated into an interaction network through two ways of evolution strategies. One is in the promoter region of the gene, which evolved a *cis* element for binding of Sox9, a transcription factor for male sex determination and spermatogenesis. Another one is in the coding region, which acquired its ability of interaction with key autophagy initiation factor Becn1. These two features endow the new gene *srag* with ability to integrate into preexisting network in autophagy regulation in testis.

The Sox9–*srag* pathway determines where the new gene functions, which is essential for Srag to exert its role in promoting autophagy specifically in testis. Both in vitro and in vivo experiments confirmed that the Sox9 binding in the *srag* promotor is key for *srag* transcription in testis. Successful evolution of the binding site for Sox9 in *srag* is an important step to integrate into preexisting gene network for the new gene. Consistent with this, the new gene *Zeus* in *Drosophila* evolved over 100 amino acid substitutions in its DNA-binding motif under positive selection. Consequently, it evolved hundreds of new gene links to build the gene networks that regulate reproduction (Chen et al. 2012). Our studies demonstrated that Sox9–*srag* gene network guided a role of Srag in testis. Previous studies in Sox9 support the findings. SOX9 was expressed in Sertoli cells and essential for testis differentiation (Kent et al. 1996; Morais da Silva et al. 1996). *Sox9* in transgenic mice with the XX chromosomes can induce testis development (Vidal et al. 2001), whereas deletion or duplication of core enhancers upstream of *SOX9* in humans (Croft et al. 2018) and deletion of a single distal enhancer of *Sox9* in mice (Gonen et al. 2018) caused sex reversal. In addition, Srag expression in Sertoli cells was regulated by Sox9. It should be pointed out that Srag was also expressed in spermatogonia, which would be regulated by other transcription factors, such as Gata1. The expression pattern of Srag will benefit regulation of autophagy, together with autophagy protein Becn1, in both Sertoli cells and spermatogonia.

Molecular network underlying autophagy initiation is not well understood. Upon autophagy induction, the ULK complex (Ulk1, Atg13, Fip200, and Atg101) is activated, which regulates the class III phosphatidylinositol 3-kinase complex (Becn1, Atg14, Vps15, Vps34, and Ambra1), then the effector of phosphatidylinositol 3-phosphate (PtdIns3P) and the WIPI proteins. For elongation of isolation membrane, ubiquitin-like conjugation complexes promote formation of ATG5–ATG12–ATG16L1, which drives the LC3B lipidation (Yu et al. 2018). During the process, RAB37, a small GTPase, can interact with ATG5 via GDP–GTP (guanosine triphosphate) cycling and promote autophagosome formation by modulating ATG5–ATG12–ATG16L1 complex assembly (Sheng et al. 2018; Song et al. 2018). Autophagy-related genes (ATGs) are evolutionarily conserved from yeast to human. Among them is Becn1, a central regulator of autophagy initiation. The C-terminal of Becn1 is evolutionarily conserved and deletion of the C-terminal showed autophagy defects (Fogel et al. 2013). Srag can interact with the C-terminal of Becn1, suggesting that the Sox9–Srag pathway can integrate into preexisting autophagy network. We speculate that conservation of the C-terminal of Becn1 presets a pose to accept new partners from newly evolved genes, such as *srag*, which generates new pathway. *srag-*transgenic zebrafish revealed an upregulated autophagy in testis, confirming the viewpoint. Indeed, cell type-specific autophagy was observed under basal or stress conditions in several tissues (Chen et al. 2014; Maday and Holzbaur 2016). Identification of the regulators of cell type-specific or sex differential autophagy has become particularly important for exploration of cellular homeostasis mechanisms in various physiological and pathological conditions (Yuan et al. 2015; Shang et al. 2020). Our study provides a better understanding of roles and molecular mechanisms of new genes through integrating into preexisting pathway in autophagy regulation in testis.

## Materials and Methods

### Animals

The *M. albus* were obtained from Wuhan area in the Yangtze River basin in China. By the microscopic analysis of their gonad sections, the phenotypic sex was verified. Wild-type zebrafish was purchased from China Zebrafish Resource Center. All the animal experiments and methods were executed in accordance with the relevant approved guidelines and regulations, as well as approved by the Ethics Committee of Wuhan University.

### Plasmid Constructs

Full-length *srag* (XP_020461511.1) was cloned into pCMV-Tag2B, pSico-3xFlag-C1, pcDNA3.0-MYC, eGFP-N1, and pET28a using *Eco*RI and *Xho*I to generate 3xFLAG-Srag, pSico-3xFlag-Srag, MYC-Srag, GFP-Srag, and His-Srag, respectively. Full-length *gata1* (NW_018128035.1), *sox9a1* (NW_018128164.1), and *sox9a2* (NW_018128272.1) were cloned into pcDNA3.0-MYC using *Eco*RI and *Xho*I to generate pCMV-*gata1*, pCMV-*sox9a1*, and pCMV-*sox9a2*, respectively. Eight truncated fragments of the *srag* promoter were amplified from *Monopterus* genomic DNA, which were double-digested with *Mlu*I and *Xho*I and cloned into the pGL3-basic vector. Site-directed mutagenesis for the Gata1- and Sox9-binding sites was performed using the primers described in supplementary table S1, Supplementary Material online. Wild-type pGL3-*srag*-5 plasmid was used as the template to construct those mutants. Full-length Becn1 (NW_018127949.1) was amplified from *Monopterus* cDNA, then cloned into pSico-Cherry-Flag and pcDNA3.0-MYC using *Eco*RI and *Xho*I to generate Cherry-Becn1 and MYC-Becn1, respectively. The Becn1 fragments consisting of MYC-Becn1 residues 1–137, 138–352, 353–447, and 138–447 were amplified using PCR primers described in supplementary table S1, Supplementary Material online. These fragments were digested with *Eco*RI and *Xho*I and ligated into pcDNA3.0-MYC. A two-step PCR-based mutagenesis method was used to construct Becn1 deletion mutants lacking the BH3, CCD, or ECD domain. Two partially overlapping fragments were amplified by primers Becn1 BH3Δ-F and MYC-Becn1-R, and MYC-Becn1-F and Becn1 BH3Δ-R using MYC-Becn1 as the template in first-step PCR. Two DNA fragments from step 1 were annealed and used as the template for second-step PCR. The primers MYC-Becn1-F and MYC-Becn1-R were used to obtain the Becn1 BH3 mutant. The resulting PCR product was cloned into pcDNA3.0-MYC to obtain MYC-Becn1-BH3Δ. The deletion mutant constructs MYC-Becn1-CCDΔ and MYC-Becn1-ECDΔ were generated in the same steps as described above. All constructs were sequenced. The primers and PCR conditions are described in supplementary table S1, Supplementary Material online.

### Antibodies

The following primary antibodies were used: Anti-ATG5 (Cat# sc-133158) was purchased from Santa Cruz Biotechnology, Dallas, TX. Anti-BECN1 (Beclin 1, autophagy related) (Cat#PD017) was purchased from MBL. Monoclonal ant-Sox9 (Lot# J0517) was from Santa Cruz Biotechnology. Anti-GAPDH (glyceraldehyde-3-phosphate dehydrogenase) (Cat# CW0100A) was from CWBIO, Beijing, China. Anti-Sox9 (# 82630S) was purchased from Cell Signaling Technology, Bossdun, MA. Monoclonal anti-Vasa (# 128306) was purchased from Gene Tex, San Antonio, TX. Anti-FLAG antibody (Cat# F3165) and monoclonal anti-LC3B (Cat# SAB4200361, produced in mouse) were from Sigma-Aldrich, St Louis, MO. Anti-MYC (Cat# 11667149001) was from Roche Applied Science, Indianapolis, IN. Anti-p62 (SQSTM1) (Cat# 18420-1-AP) antibody was from Proteintech Group, Rosemont, IL.

Secondary antibodies: Goat anti-mouse IgG (H+L), horseradish peroxidase-conjugated antibody (Cat# 31430) were from Pierce Biotechnology Company, Rockford, IL. Peroxidase-conjugated AffiniPure goat anti-mouse IgG, light chain specific (115-035-174) was from Jackson ImmunoResearch Laboratories, West Grove, PA. Peroxidase-conjugated AffiniPure F(ab')2 fragment rabbit anti-mouse IgG, Fc fragment specific (315-036-046) was from Jackson ImmunoResearch Laboratories. Peroxidase-conjugated AffiniPure mouse anti-Rabbit IgG, light chain specific (211-032-171) was from Jackson ImmunoResearch Laboratories. Peroxidase-conjugated AffiniPure F(ab')2 fragment goat anti-Rabbit IgG, Fc fragment specific (211-032-170) was from Jackson ImmunoResearch Laboratories. TRITC-conjugated ImmunoPure goat anti-rabbit IgG (H+L) (Cat# ZF-0316) and fluorescein isothiocyanate (FITC)-conjugated ImmunoPure goat anti-rabbit IgG(H+L) (Cat# ZF-0311) were purchased from Feiyi Technology, Wuhan, Hubei Province, China.

Production of anti-Srag antibody was performed in rabbit. A cDNA fragment of *srag* in *Monopterus* was cloned into pet-28a (+) to generate His-Srag fusion protein using primer sequences which are described in supplementary table S1, Supplementary Material online. The fusion protein was expressed in bacteria BL21, purified using Ni2+-NTA-agarose. Rabbits were immunized with the purified Srag protein, and high quality of antibody was purified from the antiserum with Protein-G-agarose (CW0012; CWBIO, Beijing, China) under the manufacturer’s recommended procedures.

### Analysis of New Genes

Vertebrate and invertebrate protein sequences (64,393,748) were downloaded from web (ftp://ftp.uniprot.org/pub/databases/uniprot/uniref/uniref90/), *Monopterus* protein sequences (20,456) were searched against those sequences by BlastP (*E* < 10^−10^), and 3,282 genes were identified which showed no significant BlastP hits in all organisms. After excluded the too short genes (≤65 amino acids) or the genes without start and stop codons, we supposed that 1,950 genes were the candidate orphan genes in *Monopterus*. The candidate genes were confirmed by RNA-seq data set we sequenced (GEO, GSE43649) (Zhao et al. 2018). In total, 1,533 genes were determined as new protein-coding orphan genes.

### Real-Time and Semiquantitative RT-PCR

TRIzol (15596-026, Invitrogen, Carlsbad, CA) was used to isolate total RNA, which was transcribed using a poly (T)18 primer and reverse transcriptase from the total RNAs with M-MLV (Moloney Murine Leukemia Virus-derived RTase) reverse transcriptase (M1701, Promega, Madison, WI). Semiquantitative RT-PCR was used to amplify cDNAs from gonad tissues in *Monopterus*. *hprt* (hypoxanthine guanine phosphoribosyl transferase) mRNA was amplified as an internal control. The primer sequences are described in supplementary table S1, Supplementary Material online. Platinum SYBR Green qPCR Super Mix-UDG (D01010A, Invitrogen) was used for real-time PCR amplification of *srag* in a StepOne real-time PCR system (Applied Biosystems).

### Cell Culture, Transfection, Starvation Treatments, and Dual-Luciferase Reporter Assays

HeLa and HEK293T cells were obtained from China Center for Type Culture Collection (3115CNCB00209 and 3115CNCB00266). HEK293T and HeLa cells were cultured in dulbecco's modified eagle medium (DMEM) (SH30022.01B, HyClone, Logan, UT) with 10% fatal bovine serum (FBS) (P30-330250, PAN-Biotech, Aidenbach, Germany). To establish a stable Srag-overexpression cell line, HeLa cells were transfected with the pSico-3xFlag-Srag plasmid using Lipofectamine 2000 Transfection Reagent (11668027, Invitrogen). Lentiviral vectors were cotransfected with the lentiviral packaging vectors pRSV-Rev (#12253, Addgene, MA), pMD2.G (#12259, Addgene), and pCMV-VSV-G (#8454, Addgene) into HEK293T cells using Lipofectamine TM2000. The collected supernatants were filtered through a 0.45-μm filter after transfection for 48 h and used directly to infect HeLa cells.

For starvation treatments, the cells were cultured in the medium EBSS (Cat# SH30029.02, HyClone) or HBSS (SH30030.02B, HyClone) for various times. For bafilomycin A1 treatment, Bafilomycin A1 (B1793, Sigma-Aldrich) was added in the medium for various times before harvest (Li et al. 2020).

For luciferase assays, each plasmid was transfected with 400 ng, together with 10 ng/well of pRL-TK (E2241, Promega). After transfection, luciferase activities were measured using a dual-luciferase reporter assay system (Promega) and a Modulus Single Tube Multimode Reader (Turner Biosystems, Sunnyvale, CA). Experiments were independently repeated at least three times, and the results were expressed as the means ± standard deviation (SD).

### Western Blots and Co-IP Assays

Western blots and Co-IP were performed as routine protocols (Sheng et al. 2018). Briefly, the proteins were extracted from 293T cells or samples of *Monopterus*. The whole extracts were analyzed using sodium dodecyl sulfate polyacrylamide gel electrophoresis (SDS-PAGE) and transferred to a 0.45-μm polyvinylidene fluoride (PVDF) membrane (Cat# NK0414, Roche Diagnostics). The membranes were blocked with 5% bovine serum albumin (BSA) in TBST (20 mM Tris-HCl, pH 7.5, 150 mM NaCl, 0.1% Tween-20), incubated with the antibodies overnight at 4 °C, then with the horseradish peroxidase-labeled secondary antibody. Finally, the signals were visualized by incubating membranes with the ECL kit (Millipore, Billerica, MA). Band intensity values were detected using ImageJ (version J2, NIH, MD). Data were prepared as excel files and analyzed by Microsoft excel software. To analyze protein interactions, Co-IP assays were performed in HEK293T or samples of *Monopterus*. HEK293T cells were cotransfected with related plasmid DNAs. After 48 h cells were lysed in RIPA buffer consisting of 50 mM Tris-HCl at pH 8.0, 0.15 M NaCl, 1 mM ethylenediaminetetraacetic acid, 0.5% NP-40, and a 1× protease inhibitor cocktail (Cat# 04693159001, Roche Applied Science). The cell lysates were incubated with the appropriate antibody and Protein G Agarose (Cat# 11243233001, Roche) overnight at 4 °C. The Agarose was collected by centrifugation at 4 °C and then washed five times with RIPA buffer. Bound proteins were eluted using loading buffer (50 mM Tris-HCl, 2% SDS, 1.5% mercaptoethanol, 10% glycerol, 0.01% bromophenol blue, pH 6.8) in 100 °C for 10 min and separated using 12% SDS-PAGE, followed by immunoblotting with the appropriate antibodies.

### In Situ Hybridization

*srag* cDNA was subcloned into pGEM-T Easy plasmid vector, then antisense and sense RNA probes were prepared, respectively, from subclones (linearized by restriction enzyme, *Nde*I), labeled with digoxigenin-UTP (1209256, Roche), using T7 RNA polymerase (00422456, Thermo, Rockford, IL). Gonadal tissues were cut using freezing microtome (Leica, CM1850, Wetzlar, Germany), and the sections were immediately hybridized with the probe. RNase-free container was used to make sure to keep moisture in the container during hybridization. Hybridization was performed with final concentration of DIG-labeled RNA probe at 1 μg/10 ml of hybridization buffer (usually 1:100 dilution). Hybridization buffer was denatured for 10 min at 85 °C and immediately put on ice before probe added. About 150–200 μl of hybridization solution was added onto each slide, covered slide with coverslip. Then, the slide was incubated at 70 °C overnight for probes. Hybridization signals were detected by use of the NBT/BCIP system according to the manufacturer’s instructions (11681451001, Roche).

### Immunofluorescence Analysis

Immunofluorescence analysis was performed according to our previous protocols (Yuan et al. 2015). Briefly, the tissues were embedded in paraffin and cut into a series of 2-µm sections using a slicer (Leica, Bensheim, Germany). HeLa cells were cultured on glass coverslips. At 48 h after transfection using Lipofectamine TM2000, the cells were starved in EBSS for 1 h. The cells and tissue sections were fixed with 4% paraformaldehyde for 30 min at 4 °C and antigen retrieval was performed with the AR buffer (Leica, AR9961, Germany), then permeabilized with 0.1% Triton X-100 in phosphate-buffered saline (PBS) for 20 min. After blocked in 5% BSA for at least 30 min at room temperature, the samples were incubated with primary antibody overnight at 4 °C. After washing with PBS three times, the samples were incubated with indirect immunofluorescence secondary antibody for 1 h at room temperature. The nuclei were stained with Hoechst (Cat# C1022, Beyotime Institute of Biotechnology, Jiangsu Province, China) or DAPI (#1155MG010, Biofroxx, Germany) staining solution for 5 min in the dark. Images were captured with a confocal fluorescence microscope (Olympus, FV1000, Tokyo, Japan). For colocalization analysis, plasmids with different fluorescence tag were cotransfected into HeLa cells. After 48-h transfection, the images were taken by the confocal fluorescence microscope (Olympus, FV1000). Colocalized dots were counted and data were prepared as excel files. Data were expressed as means ± SD. *T*-test was used for the statistical analysis: *P < 0.05; **P < 0.01.

### Chromatin Immunoprecipitation

ChIP was performed according to our previous study (Fu et al. 2015). Briefly, testis samples from adult *Monopterus* were cut into small pieces, and 1% formaldehyde-PBS was used for crosslinking for 15 min with constant shaking. Then, glycine was added to a final concentration of 0.125 M to terminate the formaldehyde crosslinking. The other steps were described in our previous study. The supernatant fraction chromatin was immunoprecipitated with anti-Srag, no antibody (beads only) or preimmune IgG, together with protein G PLUS-agarose (Sc-2002, Santa Cruz). DNA from the immunoprecipitated complex was PCR-amplified using primers flanking −223 to −335 bp of the *srag* genomic sequence. As a control, a 125-bp region in exon of the *srag* gene was amplified. The primer sequences are described in supplementary table S1, Supplementary Material online. The PCR fragments were cloned into the T-easy vector (A137A, Promega) and sequenced.

### Generation of Transgenic Zebrafish

Full-length *srag* sequence was cloned between *Eco*RI/*Bam*HI restriction sites in the pTol2-*actin*-2A-*egfp* vector, which was coinjected together with the Tol2 transposase mRNA. The Tol2 transposase mRNA was transcribed by the mMessage mMACHINE SP6 kit in vitro (AM1340, Ambion, Carlsbad, CA). Transposase mRNA (200 ng/µl) and the circular plasmid DNA (50 ng/µl) were coinjected into one-cell embryos of wild-type zebrafish. F1 generation was obtained by crossing of F0 founder with wild-type.

## Statistical Analysis

The data are presented as the means ± SD. Statistical comparisons were made using Student’s *t*-test when comparing two groups. One-way analysis of variance (ANOVA) with Tukey’s post hoc test was performed for comparisons among more than two groups. The analysis was performed using the statistical package IBM spss statistics software (SPSS) version 22. For significant test, a *P* value <0.05 was considered to be statistically significant as routine protocols (Hong et al. 2019).

## Ethics Statement

All animal experiments and methods were performed in accordance with the relevant approved guidelines and regulations, as well as under the approval of the Ethics Committee of Wuhan University.

## Supplementary Material

Supplementary data are available at *Molecular Biology and Evolution* online.

## Acknowledgments

This work was supported by National Key Research and Development Project (2019YFA0802500) and the National Natural Science Foundation of China (31771370, 31771487, and 31970539).

## Author Contributions

Conceptualization: H.C. and R.Z.; methodology: Y.C. and M.L.; formal analysis: Y.C. and M.L.; investigation: Y.C., F.L, X.W., D.S., J.Z., M.L., and X.X.; writing—original draft: Y.C.; writing—review & editing: Y.C. and R.Z.; supervision: H.C. and R.Z.; project administration: H.C. and R.Z.

## Supplementary Material

msaa195_Supplementary_Data
