# Supplementary material for: Srag Regulates Autophagy via Integrating into a Preexisting Autophagy Pathway in Testis
Source: Mol Biol Evol. 2020 Jul 28;38(1):128–41. doi: 10.1093/molbev/msaa195 (PMC7782868; doi:10.1093/molbev/msaa195)
Supplement: msaa195_Supplementary_Data [file msaa195_supplementary_data.pdf]

Supplementary Material for

Srag regulates autophagy via integrating into a preexisting autophagy pathway in testis

Yibin Cheng[a], Fengling Lai[a], Xin Wang[a], Dantong Shang[a], Juan Zou[a], Majing Luo[a], Xizhong Xia[a], Hanhua Cheng[a*], Rongjia Zhou[a,b*]

[a] Hubei Key Laboratory of Cell Homeostasis, College of Life Sciences, [b] Renmin Hospital of Wuhan University, Wuhan University, Wuhan 430072, China

*Corresponding authors: Professors Rongjia Zhou and Hanhua Cheng, Hubei Key Laboratory of Cell Homeostasis, College of Life Sciences, Wuhan University, Wuhan 430072, P. R. China, Fax: +86-27-68756253, E-mail: rjzhou@whu.edu.cn; hhcheng@whu.edu.cn

Supplemental 4 figures and 1 table

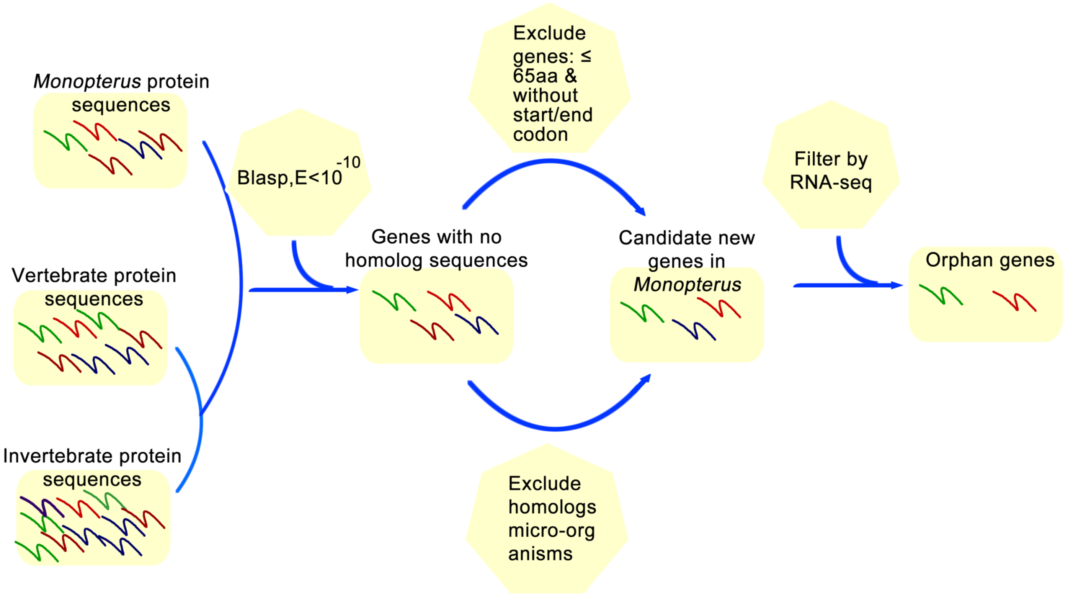

Figure S1. Identification of orphan genes in *Monopterus albus*.

Vertebrate/invertebrate protein sequences (64,393,748) were searched against *Monopterus* protein sequences (20,456) by BLASTP. Genes (3,282) with no significant Blastp hits in all organisms were identified. After excluded either too short genes or the genes without start and stop codons, 1,950 genes are the candidate orphan genes in *Monopterus*. Finally confirmed by RNA-seq datasets, new protein-coding orphan genes (1533) were obtained.

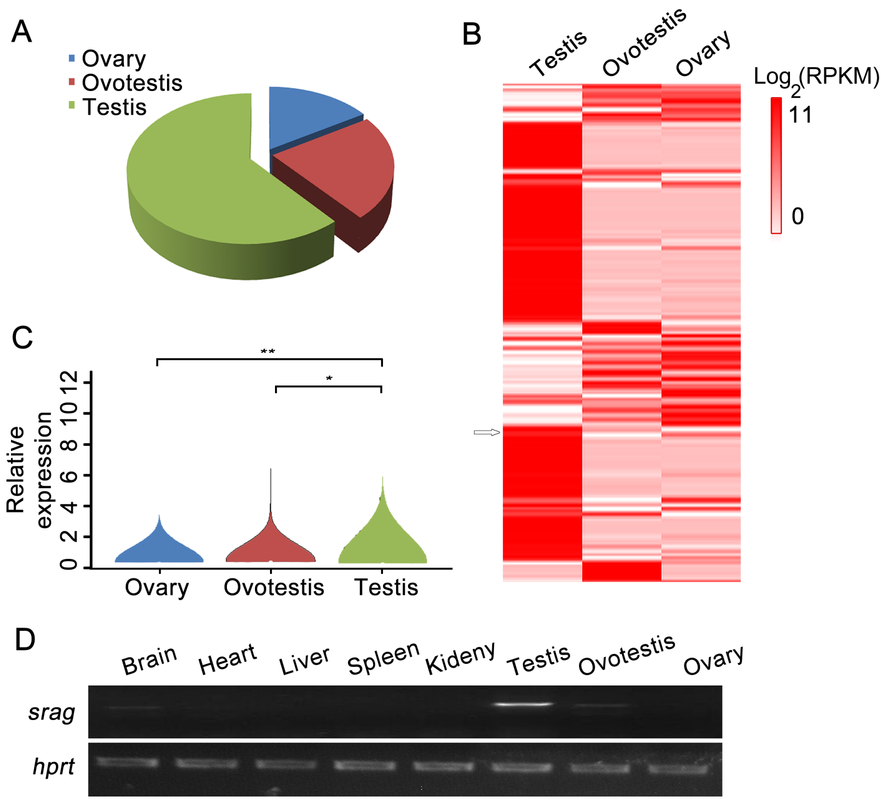

Figure S2. Identification of orphan genes in gonads.

(A) Pie chart of percentage of testis-biased orphan genes, ovary-biased orphan genes and ovotestis-biased orphan genes in the *Monopterus*. Expression levels were determined by RPKM comparison among testis, ovotestis, and ovary. (B) Heatmap of orphan genes expression levels during gonad transition. Expression levels were calculated by log2 (RPKM+1). Arrows indicate *srag*. (C) Violin plot showing the expression levels (RPKM) of all orphan genes during gonad transition (Mann-Whitney U test, *, $p < 0.05$, **, $p < 0.01$). (D) Semi-quantitative RT-PCR of *srag*. Total RNA samples were isolated from brain, heart, liver, spleen, kindey, testis, ovotestis, and ovary. RT-PCR analysis showed that *srag* was testis-biased expressed. *hprt* was used as an internal control.

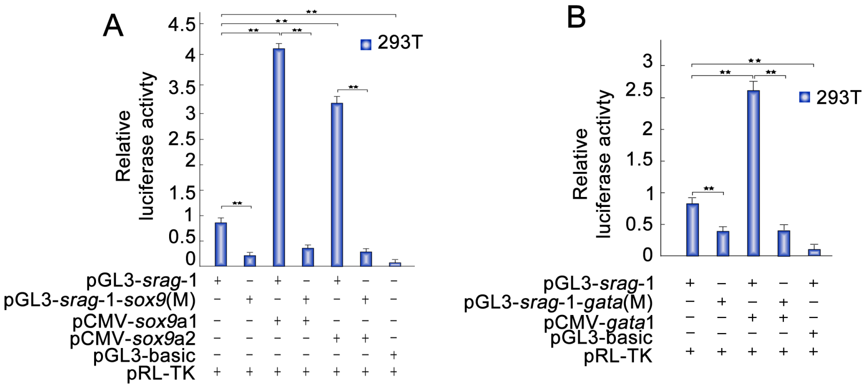

Figure S3. Sox9a1/2 and Gata1 upregulates *srag* promoter activity

(A) Sox9a1/2 overexpression up-regulated the luciferase activity of srag promoter. Sox9a1/2 transfection activated the *srag* promoter. In total, 0.32 μg pGL3-*srag*-5 or its Sox9 binding site mutant (pGL3-*srag*-1-*sox9*(mut)) was cotransfected with 0.08 μg Sox9a1/2 expression plasmids (pCMV-*sox9a1* and pCMV-*sox9a2*), as indicated. Sox9a1 and Sox9a2 overexpression increased the activity of pGL3-*srag*-1, but did not affect the activity of pGL3-*srag*-1-*sox9*(mut). (B) Gata1 overexpression up-regulated the luciferase activity of *srag* promoter. Gata1 transfection activated the *srag* promoter. In total, 0.32 μg pGL3-*srag*-1 or its Gata binding site mutant (pGL3-*srag*-1-*gata* (mut)) was cotransfected with 0.08 μg Gata1 expression plasmids (pCMV-*gata1*). Gata1 overexpression increased the activity of pGL3-*srag*-1, but did not affect the activity of pGL3-*srag*-1-*gata* (mut).

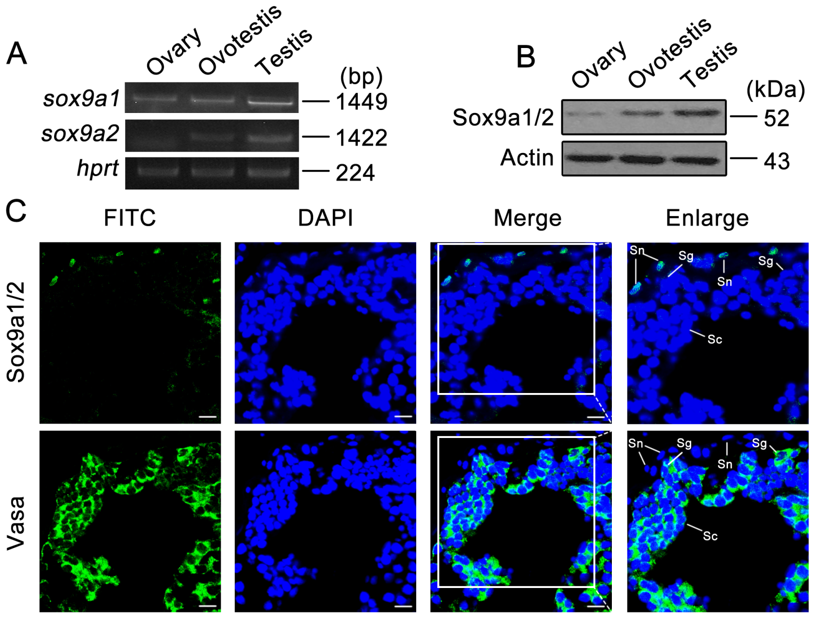

Figure S4. Expression profiles of Sox9a1/2 in *Monopterus albus*.

(A) Semi-quantitative RT-PCR of *sox9a1/2*. Total RNA samples were isolated from ovary, ovotestis, and testis. RT-PCR analysis showed that expression level of *sox9a1/2* was up-regulated during gonad transition. *hprt* was used as an internal control. (B) Western blot analysis of Sox9 protein levels in different tissues using the anti-Sox9 antibody. Total protein samples were isolated from ovary, ovotestis, and testis, with highest expression in testis. Actin was used as an internal control. (C) Immunofluorecent localization of Sox9 and Vasa proteins in testis using anti-Sox9 and anti-Vasa antibody. Sox9 was expressed in the nuclei of Sertoli cells in testis, while Vasa was mainly expressed in germ cells. The nuclei were stained by DAPI. The enlarged image originated from the region with white square. Scale bar: 10 μm,

Table S1. The primers used in the study

Genes/ Plasmid	Primer name	Primer sequence (5' - 3')
hprt	*hprt*-F	GAACAGTGACCGCTCCATCC
	hprt-R	TTGTCAGGGACCTCGAATCCT
sox9a1 (RT-PCR)	*sox9a1*-F	TGGCTGTCACTGCAGTCTTT
	sox9a1-R	CCAGCTGGCAGAAAGAAGTT
Sox9a2(RT-PCR)	*Sox9a2*-F	ACGCGTAACTTGGCACCAAAC
	Sox9a2-R	GTGGCTGTACATGTCA
pGL3-*srag*-1	pGL3-*srag*-1-F	CGCGGATCCTTTGTTGTCGCAGAGTGT
	pGL3-*srag*-1-R	CCGCTCGAGTAAAGCCTCCAGACCACC
pGL3-*srag*-2	pGL3-*srag*-2-F	CGCGGATCCTAGACGAGTCGGAGGTTT
	pGL3-*srag*-2-R	CCGCTCGAGTAAAGCCTCCAGACCACC
pGL3-*srag*-3	pGL3-*srag*-3-F	CGCGGATCCTTCTTGAAACCACCACTC
	pGL3-*srag*-3-R	CCGCTCGAGTAAAGCCTCCAGACCACC
pGL3-*srag*-4	pGL3-*srag*-4-F	CGCGGATCCGTTCCCATTGAAATACACTA
	pGL3-*srag*-4-R	CCGCTCGAGTAAAGCCTCCAGACCACC
pGL3-*srag*-5	pGL3-*srag*-5-F	CGCGGATCCCCTTGGTTCTTTGTGGAG
	pGL3-*srag*-5-R	CCGCTCGAGTAAAGCCTCCAGACCACC
pGL3- *srag*-6	pGL3-*srag*-6-F	CGCGGATCCTTTGTTGTCGCAGAGTGT
	pGL3-*srag*-6-R	CCGCTCGAGTAAAGCCTCCAGACCACC
pGL3-*srag*-7	pGL3- *srag*-7-F	CGCGGATCCTGTCGCATTTGTGAGTGT
	pGL3-*srag*-7-R	CCGCTCGAGTAAAGCCTCCAGACCACC
pGL3-*srag*-8	pGL3-*srag*-8-F	CGCGGATCTCGCAGCTTTGTTGAGTGT
	pGL3-*srag*-8-R	CCGCTCGAGTAAAGCCTCCAGACCACC
pGL3-*srag*-5-*gata*(mut)	Mut-*gata1*-S	TGCTACCGTATTTGAAAAAAAAACCGAGACAAGTGT
	Mut-*gata1*-A	ACACTTGTCTCGGTTTTTTTTTCAAATACGGTAGCA
pGL3-*srag*-5-*p53*(mut)	Mut -*p53*-S	TGTGTGATAAACCAAAAAAAATGTTTTTTTTTTT
	Mut -*p53*-A	AAAAAAAAAAACATTTTTTTTGGTTTATCACACA
pGL3-*srag*-5- *hnf* (mut)	Mut -*hnf*-S	AAACCGAGACAAGAAAAAAAAATTTTTTTGGCTGA
	Mut -*hnf*-A	TCAGCCAAAAAAATTTTTTTTTCTTGTCTCGGTTT
pGL3-*srag*-5- *sox9* (mut)	Mut -*sox9*-A	GGCTGAAAAAAAACAGCTTGCTACCGTGGTAGCA
	Mut -*sox9*-S	CGGTTTTTTTTTTTTTCGGTTTATCACACAAGTGT

pGL3-*srag*-5-*hoxa5*(mut)	Mut -*hoxa5*-A	CAGCTTGCTACCGTAAAAATGTGATAAACCGA
	Mut -*hoxa5*-S	TCGGTTTATCACATTTTTACGGTAGCAAGCTG
pCMV-*gata1*	*gata-BamH*I	CCGGAATTCATGGAGCAGTACAGTATCCT
	*gata-Sal*I	CCGCTCGAGTTAATACACTGTAACTGGCT
pCMV-*sox9a1*	eel sox9a1-*BamH*I	CCGGAATTCATGACGGAGGAACAAGACAA
	ee1 sox9a1-xho1	CCGCTCGAGGGGTCTGGTGAGCTGGGTGT
MYC-Becn1	*becn1*-eel-*EcoR*I-S	CCGGAATTCATGGAGGGCTCTAAGTCGTC
	becn1-eel-*Xho*I-A	CCGCTCGAGTTATCGGTTGTAGAACTGTG

* Sites for restriction enzymes are underlined. PCR condition: 94 °C, 30 s; 65°C -1°C, 30 s; 70°C, 2 min, 10 cycles; 94 °C, 30 s; 60°C, 30 s; 70°C, 2 min, 25 cycles
